# A Phenotyping Algorithm to Identify People With HIV in Electronic Health Record Data (HIV-Phen): Development and Evaluation Study

**DOI:** 10.2196/28620

**Published:** 2021-11-25

**Authors:** Sarah B May, Thomas P Giordano, Assaf Gottlieb

**Affiliations:** 1 School of Biomedical Informatics University of Texas Health Science Center at Houston Houston, TX United States; 2 Section of Health Services Research Department of Medicine Baylor College of Medicine Houston, TX United States; 3 Center for Innovation in Quality, Effectiveness and Safety Michael E DeBakey VA Medical Center Houston, TX United States; 4 Section of Infectious Diseases Department of Medicine Baylor College of Medicine Houston, TX United States

**Keywords:** phenotyping, algorithms, electronic health records, people with HIV, cohort identification

## Abstract

**Background:**

Identification of people with HIV from electronic health record (EHR) data is an essential first step in the study of important HIV outcomes, such as risk assessment. This task has been historically performed via manual chart review, but the increased availability of large clinical data sets has led to the emergence of phenotyping algorithms to automate this process. Existing algorithms for identifying people with HIV rely on a combination of International Classification of Disease codes and laboratory tests or closely mimic clinical testing guidelines for HIV diagnosis. However, we found that existing algorithms in the literature missed a significant proportion of people with HIV in our data.

**Objective:**

The aim of this study is to develop and evaluate HIV-Phen, an updated criteria-based HIV phenotyping algorithm.

**Methods:**

We developed an algorithm using HIV-specific laboratory tests and medications and compared it with previously published algorithms in national and local data sets to identify cohorts of people with HIV. Cohort demographics were compared with those reported in the national and local surveillance data. Chart reviews were performed on a subsample of patients from the local database to calculate the sensitivity, specificity, positive predictive value, negative predictive value, and accuracy of the algorithm.

**Results:**

Our new algorithm identified substantially more people with HIV in both national (up to an 85.75% increase) and local (up to an 83.20% increase) EHR databases than the previously published algorithms. The demographic characteristics of people with HIV identified using our algorithm were similar to those reported in national and local HIV surveillance data. Our algorithm demonstrated improved sensitivity over existing algorithms (98% vs 56%-92%) while maintaining a similar overall accuracy (96% vs 80%-96%).

**Conclusions:**

We developed and evaluated an updated criteria-based phenotyping algorithm for identifying people with HIV in EHR data that demonstrates improved sensitivity over existing algorithms.

## Introduction

### Background

The widespread adoption of electronic health records (EHRs) by health care systems over the last decade has led to an explosion in available clinical data. These databases allow researchers to retrospectively study large cohorts of patients with a specific disease or set of clinical characteristics of interest for quality improvement projects and clinical research. However, the increase in the amount of available data brings with it the need for more efficient methods of identifying patient cohorts to facilitate this research. Historically, cohorts were identified via manual chart review, a process by which a trained abstractor manually reviewed each patient record to determine their eligibility for inclusion in the study. However, this is a time- and resource-intensive process and is impractical for large EHR databases containing thousands or millions of patients.

The limitations of manual chart review have led to the emergence of phenotyping algorithms to automate the identification of patient cohorts from large data sets for a variety of conditions such as diabetes, heart disease, and asthma [1-4]. Here, we focus on automated algorithms for identifying cohorts of people with HIV in EHR databases, as such cohorts can be useful for studying engagement along every step of the HIV care continuum (diagnosis, linkage to care, retention in care, and viral suppression) [5] and identifying areas for improvement, including strategies for prevention in high-risk populations.

The earliest algorithms for identifying people with HIV used administrative data from government databases such as those comprising Medicare or Medicaid claims [6-11]. As these data sets generally contain only diagnostic (International Classification of Disease [ICD]) and procedure (Current Procedural Terminology) codes, the cohort definition algorithms for HIV developed for them rely solely on ICD codes. An example of this type of algorithm requires at least 2 outpatient ICD codes for HIV or 1 inpatient ICD code for HIV to classify a patient as having HIV [11]. The reliability of these algorithms is however limited when applied to EHR data where relying only on ICD codes can lead to misclassification of people with HIV if these codes are used incorrectly, for example, using HIV-specific codes for testing or prevention counseling. ICD codes could also be missing as would be the case if the primary reason for the encounter was not for management of HIV infection. Recent studies have sought to improve the performance of ICD code–based algorithms on EHR data by developing phenotyping algorithms that mirror the testing and diagnostic guidelines from the US Centers for Disease Control and Prevention (CDC) [12]. These algorithms use data such as laboratory test results and prescriptions for HIV-specific medications, as well as ICD codes, to identify people with HIV from EHR records [13-18], and demonstrate good sensitivity and specificity. However, they were developed using data from single health care systems or the Department of Veterans’ Affairs, which could limit their generalizability.

As stated previously, recent HIV phenotyping algorithms developed for EHR data are based on clinical steps taken to diagnose HIV infection to provide a step-by-step procedure for identifying people with HIV in the data; for example, first identify all patients with a positive HIV screening test or an ICD code for HIV, then from this set identify those with a positive confirmatory test, etc. Although these algorithms make use of the clinical information contained in the EHR to confirm HIV diagnosis, they can miss people with HIV who do not have complete documentation of their diagnostic history or do not have ICD codes for HIV documented in their records. We found this to be the case when we implemented 2 recently published HIV phenotyping algorithms that follow this model [18] in our data and identified a significant number of people with HIV who had clinical evidence of HIV infection but were missed by these algorithms.

An alternative method that potentially avoids misclassification because of missing data is to develop a set of criteria to define HIV diagnosis. Kramer et al [16] described a set of 3 criteria: (1) presence of an ICD, ninth revision (ICD-9) code for HIV; (2) a positive HIV laboratory test, defined as a positive screening test, positive Western blot, HIV viral load (VL) measurement regardless of result, or CD4 count measurement regardless of result; and (3) prescription for HIV-specific antiretroviral medications at any time. They found that requiring at least 2 of the 3 criteria to classify a patient as having HIV yielded the highest sensitivity, with minimal trade-off in positive predictive value. However, as the algorithm by Kramer et al [16] requires evidence of a VL, without relying on the values of the VL, changes in the guidelines for HIV testing and diagnosis could introduce false positives as VL measurement is increasingly used for diagnostic purposes (especially in the diagnosis of acute HIV infection) and not solely for monitoring infection and treatment [19].

### Objective

The objective of this study is to develop and validate a novel phenotyping algorithm to identify people with HIV in EHR data that is based on an updated set of clinical criteria and to capture people with HIV missed by existing algorithms.

## Methods

### Overview

We implemented and evaluated our new algorithm alongside multiple baseline algorithms for comparison in both a national, multi-institutional EHR database and a local EHR database from a single health care system. Both databases contain data collected before and after the transition from the ICD-9 to the ICD-10, as well as before and after the introduction of new HIV testing guidelines by the CDC. To evaluate the performance of the proposed algorithm, we used 2 different strategies. First, the distribution of several demographic characteristics, such as gender and race or ethnicity, is different among people with HIV than the general patient population [20]. Therefore, we compared the distribution of demographic factors of the cohorts of people with HIV who were identified with those reported by the local health department and the CDC to confirm that our algorithm identified a cohort with representative demographic characteristics. Second, we validated our algorithm in the local EHR database by performing chart review on a subsample of patients (both people with HIV and people without HIV) and calculating the sensitivity, specificity, positive predictive value, negative predictive value, and accuracy.

### Data

Our algorithm was developed and evaluated using both local and national data sets. Our national data set is Cerner HealthFacts, a deidentified EHR database containing records of 69 million unique patients from over 600 participating hospitals and clinics spanning 19 years from 1999 to 2017. Local data were derived from University of Texas (UT) Physicians, an outpatient network based in Houston, Texas. This database contains records of approximately 4 million patients between 2006 and 2020. Both data sets have undergone harmonization and normalization procedures by the Cerner organization (national data) or the UTHealth clinical data warehouse team (local data) to ensure data validity. Patient demographics (gender, race or ethnicity, marital status, and insurance), census region of clinic (for the national database), urban or rural status, diagnosis codes, results of laboratory studies, and medications were extracted from both databases for all patients aged ≥13 years. Furthermore, 13 was selected as the age threshold for inclusion in the study because testing guidelines from the CDC recommend beginning screening for HIV at the age of 13 years [19]. The use of these data in this study was approved by the UTHealth Committee for the Protection of Human Subjects.

### Baseline and HIV-Phen Phenotyping Algorithms

We implemented 4 previously published HIV phenotyping algorithms in both data sets and used them as baseline comparators for our new algorithm. The first baseline algorithm is based only on ICD codes for HIV described by Fultz et al [11]. This algorithm requires at least 2 ICD codes for HIV documented in an outpatient setting or 1 ICD code for HIV documented in an inpatient setting to classify a patient as a person with HIV. A complete list of ICD-9 and ICD-10 codes required to implement this algorithm can be found in Multimedia Appendix 1 (Table S1).

A total of 2 HIV phenotyping algorithms described by Paul et al [18] were used as the second and third baselines. The second baseline algorithm closely follows CDC testing and diagnostic guidelines and relies on laboratory results and medications to identify people with HIV. This algorithm first identified all patients with a positive HIV antibody screening test (Multimedia Appendix 1; Table S2) and then identified those in this group with a positive HIV confirmatory test (Western blot, immunofluorescence assay, or HIV-1/2 differentiation assay [MultiSpot or Geenius]; Multimedia Appendix 2 [12]) as having a confirmed HIV diagnosis. Patients who did not have a record of an HIV screening test, had a negative or indeterminate screening test, or had a positive screening test and a negative or indeterminate confirmatory test were considered to have HIV infection if they had an HIV VL>1000 copies/mL. For patients with a VL≤1000 copies/mL or an undetectable VL, their medication history was reviewed for prescriptions for antiretroviral medications for HIV treatment, which would confirm their HIV diagnosis. The lists of HIV VL test and HIV antiretroviral medications used to implement this algorithm can be seen Multimedia Appendix 2.

The third baseline algorithm is ICD code–based and begins by identifying all patients with an ICD-9 or ICD-10 code for HIV or HIV-related comorbidities (Multimedia Appendix 1; Table S1). Patients in this group with a positive HIV confirmatory test (Multimedia Appendix 2) or HIV VL >1000 copies/mL (Multimedia Appendix 2) were considered to have a confirmed HIV diagnosis. Those who did not meet these criteria were reviewed for prescriptions for HIV antiretroviral medications (Multimedia Appendix 2) to confirm HIV diagnosis.

The fourth and final baseline we implemented as a comparator for our new algorithm was the criteria-based algorithm described by Kramer et al [16]. This algorithm defines a set of 3 criteria and requires that at least 2 of the 3 criteria be met to classify a patient as having HIV. These criteria are (1) presence of an ICD-9 code for HIV, (2) a positive HIV laboratory test, defined as a positive screening test, positive confirmatory test, HIV VL measurement regardless of result, or CD4 count measurement regardless of result, and (3) prescription for HIV-specific antiretroviral medications at any time. The ICD codes needed to implement this algorithm are listed in Table S1 of Multimedia Appendix 1, HIV screening tests are listed in Table S2 of Multimedia Appendix 1, CD4 count tests are listed in Table S3 of Multimedia Appendix 1, HIV confirmatory tests are listed in Multimedia Appendix 2, and HIV VL test are listed Multimedia Appendix 2.

**Figure 1:**
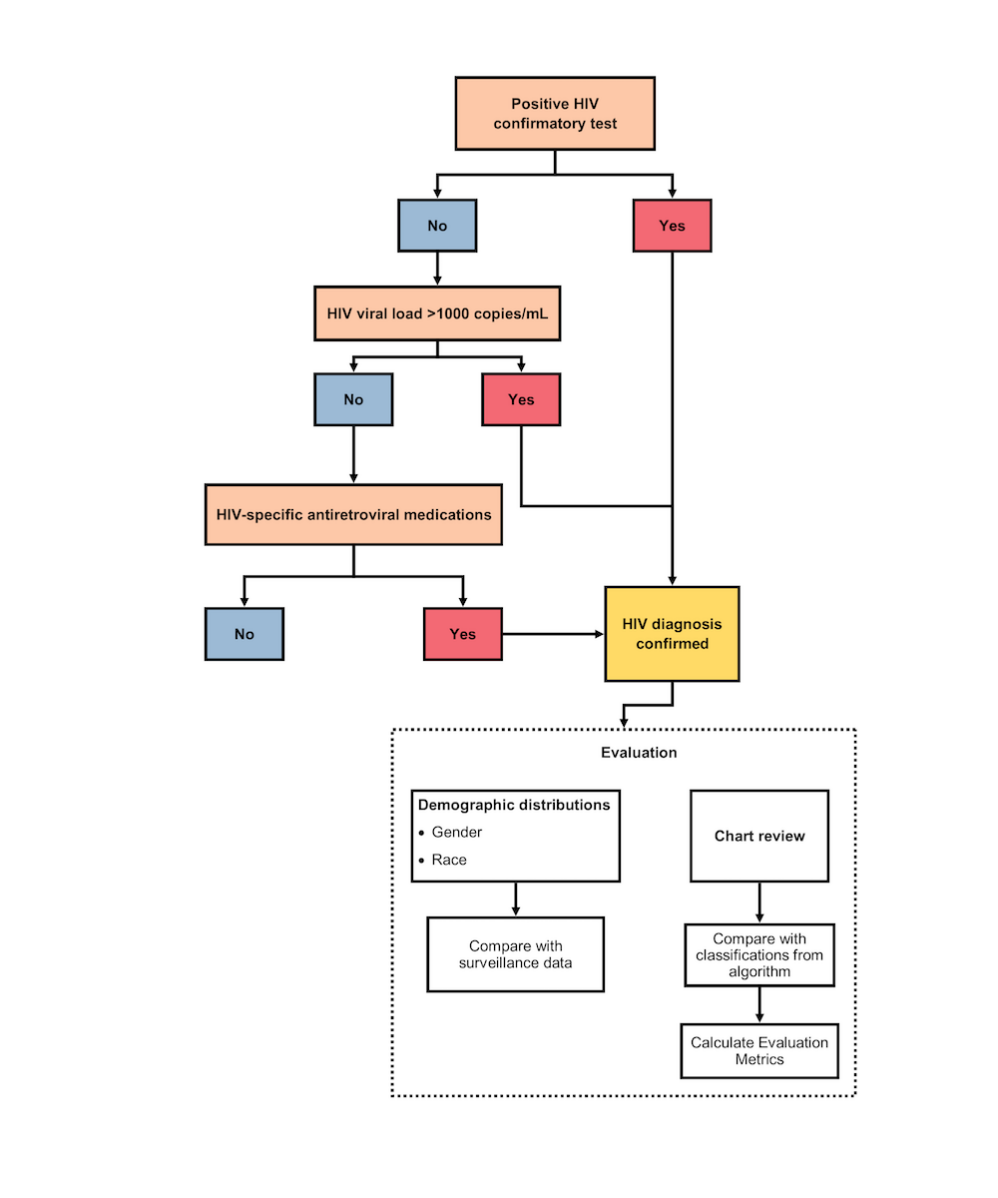
Diagram of our HIV phenotyping algorithm and evaluation framework.

Our new algorithm identifies a minimum set of clinical criteria, only one of which must be met to confirm HIV diagnosis. These criteria are a positive HIV confirmatory test, an HIV VL >1000 copies/mL, or a prescription for HIV antiretroviral medications sufficient to treat (rather than prevent) HIV as evidence of a confirmed HIV diagnosis. A decision tree representing our phenotyping algorithm is depicted in Figure 1, and the pseudocode that details the data points needed to implement this algorithm can be seen in Multimedia Appendix 2, as well as on Phenotype Knowledgebase (PheKB) [21]. Initial lists of HIV laboratory tests were generated from both the national and local databases using HIV-related keywords (*HIV*, *human immunodeficiency virus*, *rapid*, *multispot*, and *geenius*). The lists had to be generated separately for each database as laboratory test names were not standardized across institutions, and different health care systems often use different names and terminology for the tests. These lists were reviewed by a clinical domain expert (TPG) to generate the final lists of relevant laboratory tests for each data set to be included in the algorithm. In addition, a list of HIV antiretroviral medications used to treat HIV was compiled with the assistance of the same clinical domain expert. Patients being treated with only a subset of HIV antiretroviral medications that can be used to treat hepatitis B infection or for pre-exposure prophylaxis were required to have a positive confirmatory test or a VL >1000 copies/mL to be considered to have a confirmed HIV diagnosis.

### Evaluation

A total of 2 strategies were used to evaluate the performance of our algorithm: a comparison of demographic statistics to national and local statistics and a chart review to validate HIV status. First, comparisons of the demographic distributions between the cohorts of people with HIV identified using our algorithm and those with HIV included in local and national surveillance data were performed to provide evidence that our algorithm correctly identifies people with HIV from the EHR data. Demographics of the national cohort were compared with national demographic distributions of people with HIV from the HIV Surveillance Report published each year by the CDC [20], and the demographics of the local cohort were compared with demographic distributions from people with HIV in the Houston area compiled by the Houston Area Ryan White Planning Council and Houston Health Department [22].

Second, a random subsample of cases (people with HIV) and control patients was extracted from the UT Physicians data. As HIV cases are infrequent in the data, to maintain a 1:1 ratio of cases to controls, we randomly sampled patients determined as cases or controls by our algorithm. The sample size was calculated based on a 95% confidence level and a margin of error of 5%. Chart review, guided by the clinical domain expert (TPG), was then performed on this subsample to determine the HIV status of each patient by one of the researchers (SBM). On the basis of this chart review–based gold standard, the sensitivity, specificity, positive predictive value, negative predictive value, and accuracy were calculated for our algorithm. The evaluation framework is shown in Figure 1. For comparison, these metrics were also calculated for the baseline phenotyping algorithms implemented in the local EHR.

## Results

### Characteristics of People With HIV Cohorts Identified by the Algorithms

**Figure 2:**
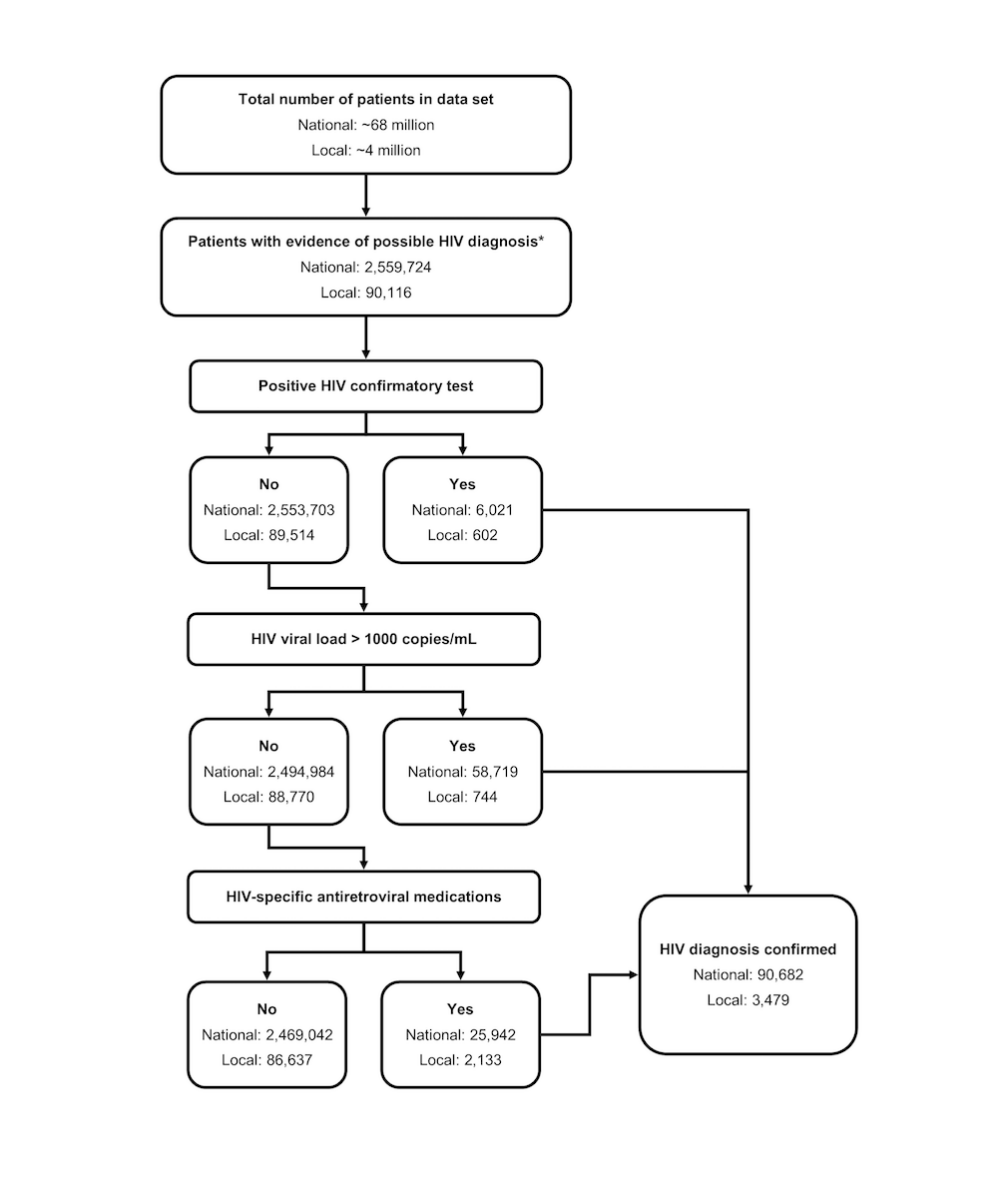
Flow diagram of patients through our algorithm for both national and local data sets. *Any International Classification of Disease code for HIV, HIV-related laboratory test performed regardless of result, or medication used to treat HIV documented in the data.

**Figure 3:**
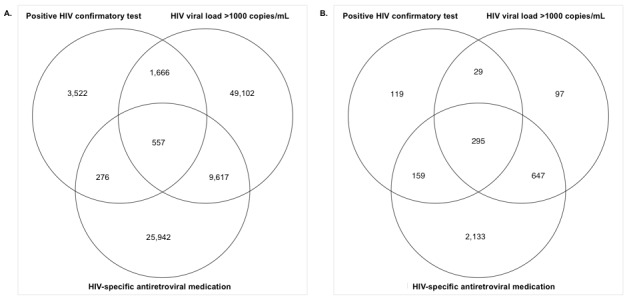
Venn diagram showing the number of patients meeting each of the criteria of our HIV phenotyping algorithm for (A) national data set, and (B) local data set.

In the national EHR data, the ICD-only baseline identified 86,066 people with HIV, the laboratory-based baseline algorithm identified 65,629 people with HIV, the ICD-based baseline algorithm identified 48,819 people with HIV, and the criteria-based baseline identified 72,443 people with HIV. In contrast, our algorithm identified 90,682 people with HIV. This represents a 5.36%, 38.17%, 85.75%, and 25.18% increase in the number of people with HIV identified in this data set over the baseline algorithms, respectively. A diagram showing the flow of patients using our algorithm is displayed in Figure 2. We examined how patients qualified as having HIV using our algorithm to identify why it resulted in the identification of more people with HIV. A Venn diagram showing the number of people with HIV identified by each criterion of the new algorithm in the national data set is shown in Figure 3A. Most of the patients in the cohort identified by our algorithm (58,719/90,682, 64.75%) were detected based on the presence of an HIV VL test result >1000 copies/mL, 48.47% (28,463/58,719) of whom did not have an ICD code for HIV or a positive HIV screening test. An additional 6021 patients were included in the cohort based on the presence of a positive HIV confirmatory test, making up 6.64% (6021/90,682) of the national cohort of people with HIV. Of these 6021 patients, 1732 (28.77%) did not have an ICD code for HIV or a positive screening test in the data, leading them to be missed by one or more of the baseline algorithms. Finally, 28.61% (25,942/90,682) were detected based on the presence of a prescription for HIV antiretroviral medications, and 35.75% (9274/25,942) of these patients did not have an ICD code for HIV or a positive screening test for HIV documented in the data. All people with HIV identified by the laboratory-based and ICD-based baseline algorithms were also identified by our new algorithm. However, there were 42,382 patients identified as people with HIV by the ICD-only baseline that were not identified by our algorithm. Conversely, our algorithm identified 46,994 people with HIV that the ICD-only baseline had not identified. The criteria-based baseline identified 22,536 patients as people with HIV not identified by our algorithm, whereas our algorithm identified 40,775 people with HIV not identified by the criteria-based baseline.

Similar results were obtained from the local database. The baseline algorithms identified 3399, 1899, 2764, and 2911 people with HIV in the local data (ICD-only, laboratory-based, ICD-based, and criteria-based baselines, respectively). This is in comparison with the 3479 people with HIV identified by our algorithm in these data (Figure 2). This represents a 2.35%, 83.20%, 25.87%, and 19.51% increase in the number of people with HIV identified by our new algorithm over the ICD-only, laboratory-based, ICD-based, and criteria-based baseline algorithms, respectively. Similar to the national cohort, all people with HIV identified by the laboratory-based and ICD-based baseline algorithms were also identified by our algorithm, whereas the ICD-only baseline identified 752 people with HIV not identified by our algorithm and the criteria-based algorithm identified 84 people with HIV not identified by our algorithm. Conversely, our algorithm identified 832 people with HIV not identified by the ICD-only baseline and 652 people with HIV not identified by the criteria-based baseline. Contrary to the national cohort, most of the local cohort (2133/3479, 61.31%) were identified by the presence of a prescription for HIV antiretroviral medications, 26.91% (574/2133) of whom did not have an ICD code for HIV or a positive HIV screening test in the data. Only 21.39% (744/3479 patients) of the local cohort were identified based on the presence of an HIV VL result >1000 copies/mL (50/744, 6.7% of whom did not have an ICD code for HIV or a positive HIV screening test), and 17.3% (602/3479 patients) based on a positive confirmatory test (18/602, 3%) of whom did not have an ICD code for HIV or a positive HIV screening test in the data). A Venn diagram showing the number of people with HIV identified by each criterion of the new algorithm in the local data set is shown in Figure 3B.

### Demographic Characteristics of People With HIV Cohorts Identified Using Our Algorithm

**Figure 4:**
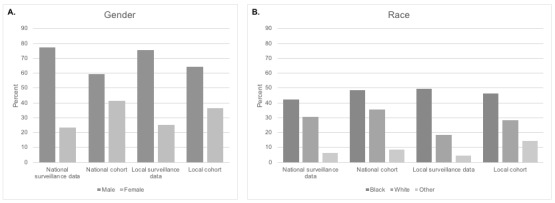
Comparison of distributions of gender and race in cohorts identified by our algorithm and national (Centers for Disease Control and Prevention) and local (Houston Health Dept) HIV surveillance data.

To validate the ability of our algorithm to identify people with HIV in both data sets, we analyzed the distributions of several demographic characteristics and compared these distributions with those seen in local and national HIV surveillance data. The cohort from our national data shares race distributions similar to those reported in national surveillance data of people with HIV: 48.43% (43,915/90,682) of people with HIV are Black and 34.69% (31,462/90,682) are White in our national data compared with 40.61% (423,304/1,042,270) Black and 29.19% (304,206/1,042,270) White in the national surveillance data (Figure 4B). Our national cohort demonstrated a higher proportion of males than females, which corresponds to the distributions seen in national surveillance data collected by the CDC [20]. However, the percentage of females in our national cohort was much higher (36,738/90,682, 40.51%; Figure 4A) than that reported nationally (245,727/1,042,270, 23.58%; Figure 4A), with a correspondingly lower percentage of males.

In the local EHR data, demographic distributions of people with HIV identified by our algorithm were compared with local surveillance statistics reported by the Houston Health Department for people with HIV in the area [22]. As with the national cohort, the racial distribution of the cohort from our local data (1589/3479, 45.67% Black; 984/3479, 28.28% White) was comparable with the racial distribution reported in local surveillance data (12,424/25,132, 49.43% Black; 4608/25,132, 18.34% White; Figure 4B). Similar to the national cohort, the local cohort was slightly more female than reported in the surveillance data (1239/3479, 35.61% females in our cohort vs 6171/25,132, 24.55% in the surveillance data; Figure 4A).

### Evaluation of Our Algorithm Compared With Baseline Algorithms in Local EHR Data

A chart review was performed on a random subsample of 360 patients in the local data set to evaluate the performance of our HIV phenotyping algorithm compared with the baseline algorithms. The sensitivity of our algorithm was 98%, representing a substantial increase in sensitivity over the laboratory-based baseline algorithm (56%) and ICD-only baseline algorithm (86%), as well as a moderate increase over the ICD-based baseline algorithm (90%) and criteria-based baseline algorithm (92%). In addition, our algorithm demonstrated an increase in overall accuracy over 3 of the 4 baselines (HIV-Phen, 96%; ICD-based baseline, 95%; laboratory-based baseline, 80%; ICD-based baseline, 95%). However, these gains were accompanied by a decrease in the specificity of our algorithm compared with the baseline algorithms. Side-by-side comparisons of these results are presented in Table 1.

**Table 1:** Evaluation results^a^.

Algorithm	Source	Sensitivity	Specificity	Positive predictive value	Negative predictive value	Accuracy	Results			
							C+		C−	
							A+	A −	A+	A −
ICD^b^-only baseline	Fultz et al [11]	0.86	0.99	0.99	0.89	0.93	143	23	1	193
Laboratory-based baseline	Paul et al [18]	0.56	*1.0* ^c^	*1.0*	0.73	0.80	93	73	0	194
ICD-based baseline	Paul et al [18]	0.90	*1.0*	*1.0*	0.92	0.95	149	17	0	194
Criteria-based baseline	Kramer et al [16]	0.92	0.99	0.99	0.93	*0.96*	152	14	1	193
HIV-Phen	N/A^d^	*0.98*	0.95	0.95	*0.98*	*0.96*	162	4	9	185

^a^Results of the evaluation of the baseline algorithms and our new clinical criteria-based algorithm on a subsample of 360 patients from the local database. Classification of the patients is shown on the right side of the table (Results) broken down by the results of chart review (C+ or C−) and algorithm classification (A+ or A−).

^b^ICD: International Classification of Disease.

^c^Results are italicized for the algorithm with the highest value for each metric.

^d^N/A: not applicable.

## Discussion

### Principal Findings

We developed a novel HIV phenotyping algorithm, HIV-Phen, that relies solely on laboratory and medication data and requires only 1 of 3 clinical criteria to be met to identify people with HIV: positive HIV confirmatory test, HIV VL>1000 copies/mL, or prescription of HIV antiretrovirals for the treatment of HIV. This algorithm was developed to address a significant portion of people with HIV that were missed by previously published algorithms in both our local and national data sets. Our new algorithm is able to identify up to 85.75% more people with HIV in our data and demonstrates improved sensitivity over the baseline comparators with modest trade-off in specificity.

We found that these people with HIV were missed by the baseline algorithms because a number of people with HIV had laboratory or prescription evidence to confirm HIV diagnosis but did not have ICD codes for HIV documented in their medical records. Furthermore, patients often visit providers from multiple health care systems for care. Owing to this, in a given EHR, some people with HIV might not have all the laboratory information needed to confirm HIV diagnosis according to algorithms that mimic clinical testing and diagnostic guidelines such as the laboratory-based baseline algorithm implemented here. This leads such algorithms to misclassify people with HIV. Our algorithm was implemented and compared across both national and local EHR databases containing data spanning large time scales, including both ICD-9-CM and ICD-10 billing codes, as well as various HIV testing technologies and guidelines. In addition to differences in geographic coverage, the 2 data sets used in this study are very different in composition with the local data containing mostly outpatient data and the national data containing predominantly inpatient as well as outpatient data. The fact that our new algorithm performs well in both data sets supports its portability across EHRs from different sources.

Our algorithm demonstrates a marked improvement in sensitivity over the baseline algorithms and a small increase in accuracy in the local data. However, these gains come with small decreases in specificity. Our algorithm resulted in 9 false positives, or people who were identified as people with HIV by the algorithm when they did not have HIV. Most patients in this group were falsely identified as having HIV because they were prescribed postexposure prophylaxis (PEP) because of HIV exposure. Unlike patients on pre-exposure prophylaxis or hepatitis B virus treatment, these patients are more difficult to identify and exclude because PEP consists of a full HIV treatment regimen. As PEP is only taken for a single short period (typically 30 days) after exposure, prescription duration and count could be considered in the algorithm to correctly identify these individuals. However, this could exclude people with HIV whose infection is not being managed by a provider in the system, who are nonadherent, or have fallen out of care. Another source of false positives was variations in clinical practices, that is, patients who received prescriptions for antiretrovirals on the same day as a screening test that came back negative.

Our algorithm falsely classified 4 patients as negative when they had HIV infection. This was largely because of HIV infection only being mentioned in the text of clinical notes for these individuals and no laboratories or medications for HIV listed in their records. Owing to this, they were also misclassified by the laboratory-based baseline algorithm. In addition, these patients did not have ICD codes in the EHR and were thus, falsely classified as negative by the other baseline algorithms that make use of these codes. Out of the 4 false negatives, 1 had a detectable HIV VL, but it was <1000 copies/mL, which is not considered sufficient clinical evidence to confirm HIV diagnosis by our criteria. We chose 1000 copies/mL as the cutoff for confirming HIV to accommodate the changes over the years in the sensitivity of the VL test, as over the time frame of our data, the lower limit of detection of the VL test has gone from 400 copies/mL to 48 copies/mL to 20 copies/mL. Running our algorithm in the local data using VL thresholds of 400, 48, and 20 copies/mL increases the number of people with HIV identified by less than 1% over the 1000 copies/mL threshold, and only 1 additional patient was identified as positive in the evaluation subsample. This was the patient with a detectable VL <1000 copies/mL that was falsely classified as negative previously. Running the same analysis in the national data increases the number of people with HIV identified by 1.5%, 7%, and 10% with VL thresholds of 400, 48, and 20 copies/mL, respectively. However, a threshold of >1000 copies/mL reduces the possibility of false positivity in distinguishing acute HIV infection from a false positive screening test when the differentiation assay is negative.

As further evidence of the accuracy of our new algorithm, we found good agreement in most demographic trends with HIV surveillance data in the cohorts from both the local and national data. However, we observed a higher percentage of women in both the national and local cohorts than that reported in the surveillance data. In the local data set, this was observed in cohorts resulting from our algorithm and the baseline algorithms. Given this, and as this is a cohort derived from clinical data rather than surveillance data, the higher percentage of women could be because of differences in the characteristics of patients who use the UT Physicians network for health care compared with the general population of people with HIV in the Houston area. As most people with HIV in the local cohort were identified because they have a prescription for antiretrovirals but lack HIV laboratory data, we speculate that many of the people with HIV identified are accessing specialty care in the UT Physicians system for other conditions and their HIV infection is being managed elsewhere. Furthermore, studies have shown that women are more likely to consult a physician than men and are more likely to have health insurance and a regular source for health care [23-26], which could potentially drive the discrepancy in gender distribution between the UT Physician population and the Houston-area surveillance data.

A discrepancy in gender distribution was also observed between the national cohort and the national HIV surveillance data. On examining the distribution of gender by census region in our national cohort, we found that the gender distribution of people with HIV from the West and Midwest align very well with the national surveillance data distribution; however, people with HIV from the South and Northeast have a much higher percentage of women than reported in the national surveillance data. Most people with HIV identified by our algorithm resided in these regions. Demographic distributions among people with HIV are not uniform across the country, and regional variations exist; for example, heterosexual transmission is a more predominant risk factor in the South, which results in a higher percentage of people with HIV who are women in this region [20]. These regional variations and the characteristics of people who regularly interact with the health care system mentioned previously could be partly responsible for the differences we observed in gender distribution in the national cohort compared with the national surveillance data.

In both national and local data, the number of Hispanic patients was not accurately captured. This is likely because of differences in the way this information is collected across systems, that is, as a single race or ethnicity variable or as separate race and ethnicity variables. In our data, this likely leads to the number of White patients being overestimated in the data and Hispanic patients being underestimated, which may explain the discrepancies observed between our national and local people with HIV cohorts and national and local surveillance data.

### Limitations

Our study has several limitations. First, although the national EHR data set is very large and contains records of millions of patients from across the country, it is deidentified, and thus, lacks information such as clinical notes and identifiers that could be linked to other data sources or used to validate algorithms against medical record reviews. This information could provide a better understanding of why so many people with HIV in this data lack ICD codes for HIV. Second, the national data are aggregated from multiple clinics across the country and mapped to standard ontologies, such as Logical Observation Identifiers Names and Codes for laboratory tests by Cerner. Errors in the mapping led to ambiguity in the data; for example, tests that were mapped to HIV screening test but had values with ranges that suggested they were VL measurements. Third, although the national data do provide a nongovernmental national sample of patients, it is limited to clinics that have implemented Cerner EHRs, which could introduce bias. Finally, as laboratory test names are not standardized and different clinics use different names and terminology, lists of HIV laboratories must be generated for each data set on which the algorithm is run, which is a time-consuming process. A consistent mapping to a standardized ontology, such as Logical Observation Identifiers Names and Codes, is needed to fully automate this part of the algorithm.

### Conclusions

We have developed and evaluated HIV-Phen, a novel HIV phenotyping algorithm, to identify people with HIV in EHR data that greatly improves sensitivity over previously published algorithms. In addition, we have shown that a significant proportion of people with HIV in 2 clinical data sets do not have ICD codes for HIV and are thus missed by phenotyping algorithms that rely on this information. This work will positively impact future HIV research as our new algorithm can be applied to both single and multi-institutional data sets to accurately identify complete cohorts of people with HIV to facilitate multiple types of studies. Furthermore, this work seeks to provide a blueprint for the implementation of our algorithm to assist other researchers in the identification of their cohorts. Although the elucidation of algorithms to accurately identify specific cohorts of patients is important, further work is needed to define standard mappings of laboratory tests, medications, and other information to increase the ease and speed with which these algorithms can be implemented across different data sets.

## Appendix

Multimedia Appendix 1 Features required for baseline algorithms.

Multimedia Appendix 2 HIV phenotyping algorithm (HIV-Phen) pseudocode.

## Abbreviations

CDC: Centers for Disease Control and Prevention
EHR: electronic health record
ICD: International Classification of Disease
PEP: postexposure prophylaxis
UT: University of Texas
VL: viral load
