# Supplementary material for: A Phenotyping Algorithm to Identify People With HIV in Electronic Health Record Data (HIV-Phen): Development and Evaluation Study
Source: JMIR Form Res. 2021 Nov 25;5(11):e28620. doi: 10.2196/28620 (PMC8727048; doi:10.2196/28620)
Supplement: Multimedia Appendix 1 [file formative_v5i11e28620_app1.docx]

**Table S1**: ICD-9 and ICD-10 codes for HIV or HIV-related comorbidity

| **Type** | **Code** | **Description** |
| --- | --- | --- |
| ICD-9 | 042 | Human immunodeficiency virus (HIV) disease |
| ICD-9 | 042.0 | HIV and specific infection |
| ICD-9 | 042.1 | HIV causing other infection |
| ICD-9 | 042.2 | HIV with neoplasm |
| ICD-9 | 042.9 | Unspecified acquired immunodeficiency syndrome (AIDS) |
| ICD-9 | 043 | HIV causing condition necrotizing enterocolitis (NEC) |
| ICD-9 | 043.0 | HIV lymphadenopathy |
| ICD-9 | 043.1 | HIV causing central nervous system (CNS) disease |
| ICD-9 | 043.2 | HIV causing other disorders involving the immune mechanism |
| ICD-9 | 043.3 | HIV causing disease NEC |
| ICD-9 | 043.9 | AIDS-related complex not otherwise specified (NOS) |
| ICD-9 | 044 | Other HIV infection |
| ICD-9 | 044.0 | HIV with acute infection |
| ICD-9 | 044.9 | HIV infection NOS |
| ICD-9 | 079.53 | HIV, type 2 |
| ICD-9 | 795.78 | Positive serologic findings; HIV |
| ICD-9 | V08 | Asymptomatic HIV infection status |
| ICD-9 | 795.71 | Nonspecific serologic evidence of HIV |
| ICD-10 | B20 | HIV disease resulting in infectious and parasitic diseases |
| ICD-10 | B20.0 | HIV disease resulting in mycobacterial infection/tuberculosis |
| ICD-10 | B20.1 | HIV disease resulting in other bacterial infections |
| ICD-10 | B20.2 | HIV disease resulting in cytomegaloviral disease |
| ICD-10 | B20.3 | HIV disease resulting in other viral infections |
| ICD-10 | B20.4 | HIV disease resulting in candidiasis |
| ICD-10 | B20.5 | HIV disease resulting in other mycoses |
| ICD-10 | B20.6 | HIV disease resulting in PJP/PCP |
| ICD-10 | B20.7 | HIV disease resulting in multiple infections |
| ICD-10 | B20.8 | HIV disease resulting in other infectious and parasitic diseases |
| ICD-10 | B20.9 | HIV disease resulting in infection NOS |
| ICD-10 | B21 | HIV disease resulting in malignant neoplasms |
| ICD-10 | B21.0 | HIV disease resulting in Kaposi sarcoma |
| ICD-10 | B21.1 | HIV disease resulting in Burkitt lymphoma |
| ICD-10 | B21.2 | HIV disease resulting in other types of non-Hodgkin lymphoma |
| ICD-10 | B21.3 | HIV disease resulting in other malignant neoplasms of lymphoid, hematopoietic, and related tissue |
| ICD-10 | B21.7 | HIV disease resulting in multiple malignant neoplasms |
| ICD-10 | B21.8 | HIV disease resulting in other malignant neoplasms |
| ICD-10 | B21.9 | HIV disease resulting in unspecified malignant neoplasms |
| ICD-10 | B22 | HIV disease resulting in other specified diseases |
| ICD-10 | B22.0 | HIV disease resulting in encephalopathy/dementia |
| ICD-10 | B22.1 | HIV disease resulting in lymphoid interstitial pneumonitis |
| ICD-10 | B22.2 | HIV disease resulting in wasting syndrome/failure to thrive |
| ICD-10 | B22.7 | HIV disease resulting in multiple diseases classified elsewhere |
| ICD-10 | B23 | HIV disease resulting in other conditions |
| ICD-10 | B23.0 | Acute HIV infection syndrome |
| ICD-10 | B23.1 | HIV disease resulting in (persistent) generalized lymphadenopathy |
| ICD-10 | B23.2 | HIV disease resulting in hematological and immunological abnormalities, not elsewhere classified |
| ICD-10 | B23.8 | HIV disease resulting in other specified conditions |
| ICD-10 | B24 | Unspecified HIV disease |
| ICD-10 | Z21 | Asymptomatic HIV infection status |

**Table S2**: LOINC codes and associated lab procedure names of HIV antibody screening tests

| **LOINC Code** | **Lab Procedure Name** |
| --- | --- |
| 42600-7 | HIV 1+2 Ab [Presence] in Unspecified specimen by Immunoassay |
| 7918-6 | HIV 1+2 Ab [Presence] in Serum |
| 33866-5 | HIV 1 Ab [Presence] in Capillary blood by Immunoassay |
| 48345-3 | HIV 1+O+2 Ab [Presence] in Serum or Plasma |
| 43009-0 | HIV 1+2 IgG Ab [Presence] in Serum |
| 56888-1 | HIV 1+2 Ab+HIV1 p24 Ag [Presence] in Serum or Plasma by Immunoassay |
| 9665-1 | HIV 1 p24 Ag [Units/volume] in Serum |
| 9821-0 | HIV 1 p24 Ag [Presence] in Serum |
| 80203-3 | HIV 1 and 2 Ab [Identifier] in Serum, Plasma or Blood by Rapid immunoassay |
| 33807-9 | HIV 2 IgG Ab [Presence] in Serum |
| 51786-2 | HIV 2 Ab Signal/Cutoff in Serum or Plasma by Immunoassay |
| 44607-0 | HIV 1 [Interpretation] in Serum or Plasma by Immunoassay |
| 22356-0 | HIV 1 Ab [Units/volume] in Serum |
| 29893-5 | HIV 1 Ab [Presence] in Serum or Plasma by Immunoassay |
| 49905-3 | HIV 1 Ab [Presence] in Unspecified specimen by Rapid immunoassay |
| 49483-1 | HIV 1 [Interpretation] in Serum or Plasma by Immunoassay Narrative |
| 31201-7 | HIV 1+2 Ab [Presence] in Serum or Plasma by Immunoassay |
| 48346-1 | HIV 1+O+2 Ab [Units/volume] in Serum or Plasma |
| 58900-2 | HIV 1+2 Ab+HIV1 p24 Ag [Units/volume] in Serum or Plasma by Immunoassay |
| 43010-8 | HIV 1+2 Ab [Presence] in Unspecified specimen |
| 85037-0 | HIV 1 and 2 Ab and HIV 1 p24 Ag panel – Serum or Plasma by Immunoassay |
| 7917-8 | HIV 1 Ab [Presence] in Serum |
| 30361-0 | HIV 2 Ab [Presence] in Serum or Plasma by Immunoassay |
| 22358-6 | HIV 2 Ab [Units/volume] in Serum |
| 22357-8 | HIV 1+2 Ab [Units/volume] in Serum |
| 6431-1 | HIV identified in Unspecified specimen by Organism specific culture |
| 5223-3 | HIV 1+2 Ab [Units/volume] in Serum or Plasma by Immunoassay |
| 35437-3 | HIV 1 Ab [Presence] in Saliva (oral fluid) by Immunoassay |
| 18396-2 | HIV 1 p24 Ag [Presence] in Serum or Plasma by Immunoassay |
| 24012-7 | HIV 1 Ag [Presence] in Serum |
| 42768-2 | HIV 1 and 2 Ab [Interpretation] in Serum Narrative |
| 73905-2 | HIV 1+2 IgG Ab [Presence] in Serum or Plasma by Rapid immunoassay |
| 41144-7 | HIV 1 Ab [Presence] in Saliva (oral fluid) |
| 7919-4 | HIV 2 Ab [Presence] in Serum |
| 75666-8 | HIV 1+2 Ab and HIV1 p24 Ag [Identifier] in Serum, Plasma or Blood by Rapid Immunoassay |

**Table S3**: LOINC codes and associated lab procedure names of HIV antibody screening tests

| **LOINC Code** | **Lab Procedure Name** |
| --- | --- |
| 42600-7 | HIV 1+2 Ab [Presence] in Unspecified specimen by Immunoassay |
| 7918-6 | HIV 1+2 Ab [Presence] in Serum |
| 33866-5 | HIV 1 Ab [Presence] in Capillary blood by Immunoassay |
| 48345-3 | HIV 1+O+2 Ab [Presence] in Serum or Plasma |
| 43009-0 | HIV 1+2 IgG Ab [Presence] in Serum |
| 56888-1 | HIV 1+2 Ab+HIV1 p24 Ag [Presence] in Serum or Plasma by Immunoassay |
| 9665-1 | HIV 1 p24 Ag [Units/volume] in Serum |
| 9821-0 | HIV 1 p24 Ag [Presence] in Serum |
| 80203-3 | HIV 1 and 2 Ab [Identifier] in Serum, Plasma or Blood by Rapid immunoassay |
| 33807-9 | HIV 2 IgG Ab [Presence] in Serum |
| 51786-2 | HIV 2 Ab Signal/Cutoff in Serum or Plasma by Immunoassay |
| 44607-0 | HIV 1 [Interpretation] in Serum or Plasma by Immunoassay |
| 22356-0 | HIV 1 Ab [Units/volume] in Serum |
| 29893-5 | HIV 1 Ab [Presence] in Serum or Plasma by Immunoassay |
| 49905-3 | HIV 1 Ab [Presence] in Unspecified specimen by Rapid immunoassay |
| 49483-1 | HIV 1 [Interpretation] in Serum or Plasma by Immunoassay Narrative |
| 31201-7 | HIV 1+2 Ab [Presence] in Serum or Plasma by Immunoassay |
| 48346-1 | HIV 1+O+2 Ab [Units/volume] in Serum or Plasma |
| 58900-2 | HIV 1+2 Ab+HIV1 p24 Ag [Units/volume] in Serum or Plasma by Immunoassay |
| 43010-8 | HIV 1+2 Ab [Presence] in Unspecified specimen |
| 85037-0 | HIV 1 and 2 Ab and HIV 1 p24 Ag panel – Serum or Plasma by Immunoassay |
| 7917-8 | HIV 1 Ab [Presence] in Serum |
| 30361-0 | HIV 2 Ab [Presence] in Serum or Plasma by Immunoassay |
| 22358-6 | HIV 2 Ab [Units/volume] in Serum |
| 22357-8 | HIV 1+2 Ab [Units/volume] in Serum |
| 6431-1 | HIV identified in Unspecified specimen by Organism specific culture |
| 5223-3 | HIV 1+2 Ab [Units/volume] in Serum or Plasma by Immunoassay |
| 35437-3 | HIV 1 Ab [Presence] in Saliva (oral fluid) by Immunoassay |
| 18396-2 | HIV 1 p24 Ag [Presence] in Serum or Plasma by Immunoassay |
| 24012-7 | HIV 1 Ag [Presence] in Serum |
| 42768-2 | HIV 1 and 2 Ab [Interpretation] in Serum Narrative |
| 73905-2 | HIV 1+2 IgG Ab [Presence] in Serum or Plasma by Rapid immunoassay |
| 41144-7 | HIV 1 Ab [Presence] in Saliva (oral fluid) |
| 7919-4 | HIV 2 Ab [Presence] in Serum |
| 75666-8 | HIV 1+2 Ab and HIV1 p24 Ag [Identifier] in Serum, Plasma or Blood by Rapid Immunoassay |
