# Supplementary material for: A Phenotyping Algorithm to Identify People With HIV in Electronic Health Record Data (HIV-Phen): Development and Evaluation Study
Source: JMIR Form Res. 2021 Nov 25;5(11):e28620. doi: 10.2196/28620 (PMC8727048; doi:10.2196/28620)
Supplement: Multimedia Appendix 2 [file formative_v5i11e28620_app2.docx]

**Human Immunodeficiency Virus (HIV)**

**Phenotyping Algorithm Pseudocode**

**BACKGROUND**

Human Immunodeficiency Virus (HIV) is a retrovirus that attacks the immune system, specifically CD4+ T-cells. If left untreated, infection causes patients to become severely immunocompromised culminating in Stage 3 infection or Acquired Immunodeficiency Syndrome (AIDS). With the development of HIV-specific antiretroviral medications, HIV infection has transformed from a terminal illness to a chronic condition, and with treatment, people with HIV have life expectancies that mirror their non-infected counterparts. Consistent access and adherence to treatment is necessary for people with HIV to remain healthy and to reduce transmission to others. For more information, see cdc.gov/hiv/basics/whatishiv.html.

**ALGORITHM DATA ELEMENTS**

This algorithm requires the following data elements:

- Patient age
- Laboratory test results
  - HIV confirmatory test results
  - HIV viral load measurement test results
- HIV-specific antiretroviral medications

Listings of laboratory tests derived from our datasets and medications required for the algorithm can be found in the appendices at the end of this document.

**CASES**

People with HIV (cases) are identified by first applying the inclusion criteria and then excluding patients who meet any of the exclusion criteria.

**Case Inclusion Criteria:**

Potential cases must meet *any one* of the following inclusion criteria:

1. Has ever had a positive result on an HIV confirmatory test (see Appendix A)
2. Has ever had an HIV viral load measurement (Appendix B) greater than 1000 copies/mL
3. Has ever been prescribed an antiretroviral medication used to treat HIV infection (Appendix C)

**Case Exclusion Criteria:**

Patients are excluded from the cohort of potential cases if they meet *any* of the following criteria:

1. Under 13 years of age at the time of assessment
2. Has been prescribed any antiretroviral medication used for HIV pre-exposure prophylaxis (PrEP) or to treat Hepatitis B (HBV) infection (Appendix D) and meets *all of* the following criteria
   - 1. Absence of a prescription for other HIV-specific antiretroviral medications listed in Appendix C
     2. Does not meet inclusion criteria I.1 and I.2

**CONTROLS**

Controls are identified by applying the control inclusion criteria and then excluding patients who meet any of the control exclusion criteria.

**Control Inclusion Criteria:**

Potential controls must meet *any one* of the following criteria:

1. Does not meet any of the case inclusion criteria
2. Excluded from the case cohort due to case exclusion criteria E.2

**Control Exclusion Criteria:**

Patients are excluded from the cohort of potential controls if they meet any of the following criteria:

1. Under 13 years of age at the time of assessment

**FLOW DIAGRAM**


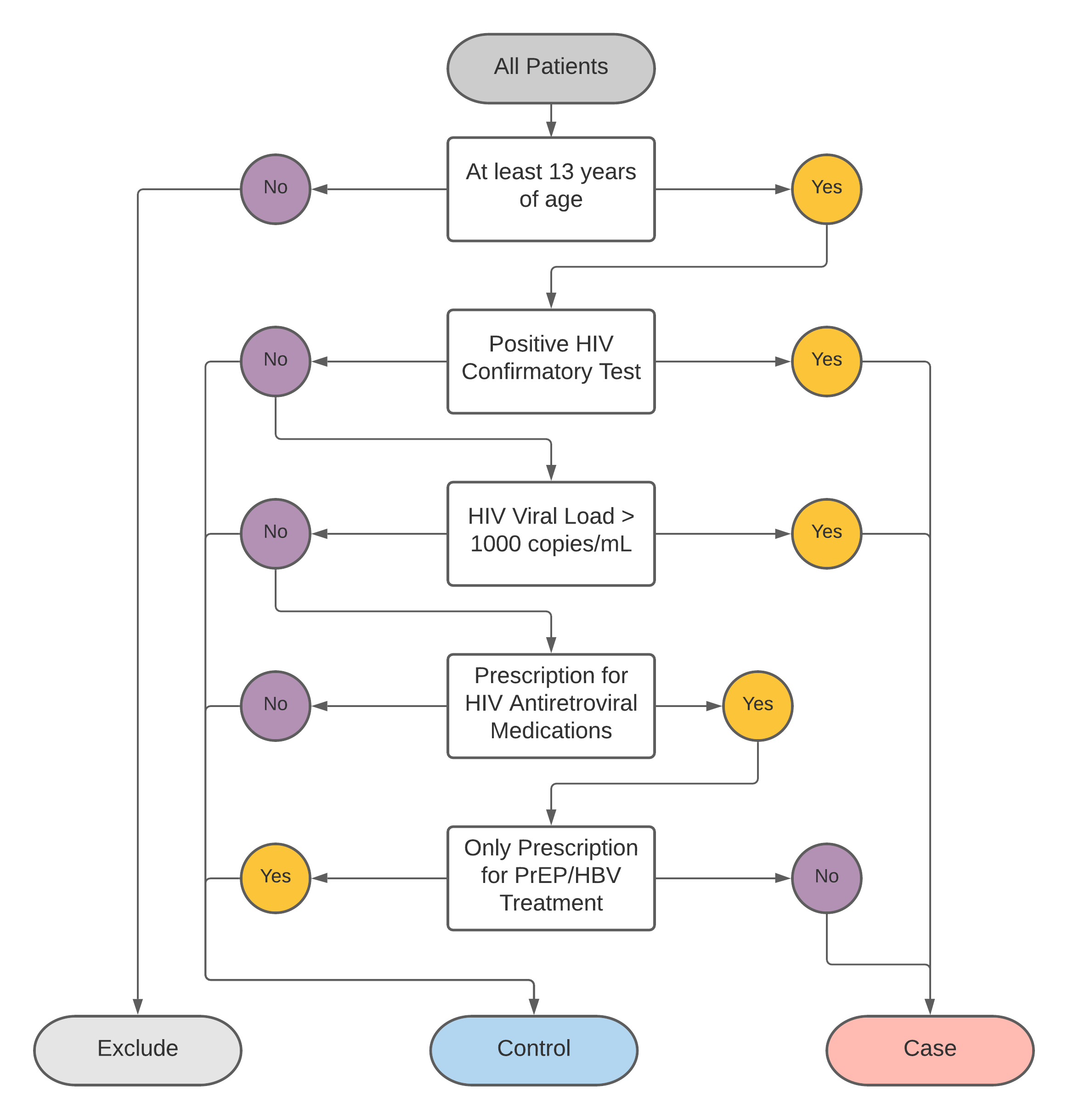


**Appendix A**

**HIV Confirmatory Lab Tests**

To make a diagnosis of HIV infection, a patient who tests positive on an HIV screening test must subsequently test positive on and HIV confirmatory test (CDC screening guidelines: <http://dx.doi.org/10.15620/cdc.23447>). These confirmatory tests include Western blot, immunofluorescence assays, and HIV-1/2 differentiation assays. Table 1 provides a list of LOINC codes and associated names of HIV confirmatory tests.

| **Table 1: HIV Confirmatory Tests** | |
| --- | --- |
| **LOINC Code** | **Lab Procedure Name** |
| 14092-1 | HIV 1 Ab [Presence] in Serum by Immunofluorescence |
| 5221-7 | HIV 1 Ab [Presence] in Serum or Plasma by Immunoblot |
| 33806-1 | HIV 2 IgG Ab [Presence] in Serum by Immunoblot |
| 5225-8 | HIV 2 Ab [Presence] in Serum by Immunoblot |
| 44873-8 | HIV 1+2 Ab [Presence] in Serum by Immunoblot |
| 13499-9 | HIV 1 Ab band pattern [Interpretation] in Serum by Immunoblot |
| 69668-2 | HIV 1 and 2 Ab [Identifier] in Serum or Plasma by Rapid immunoassay |
| 40732-0 | HIV 1 IgG Ab [Presence] in Serum by Immunoblot |

**Appendix B**

**HIV Viral Load Laboratory Tests**

Table 2 provides a list of LOINC codes and associated names of HIV viral load laboratory tests.

| **Table 2: HIV Viral Load Tests** | |
| --- | --- |
| **LOINC Code** | **Lab Procedure Name** |
| 10351-5 | HIV 1 RNA [Units/volume] (viral load) in Serum or Plasma by Probe with amplification |
| 23876-6 | HIV 1 RNA [Units/volume] (viral load) in Plasma by Probe with signal amplification |
| 20447-9 | HIV 1 RNA [#/volume] (viral load) in Serum or Plasma by NAA with probe detection |
| 49890-7 | HIV 1 RNA [Log #/volume] (viral load) in Unspecified specimen by NAA with probe detection |
| 29539-4 | HIV 1 RNA [Log #/volume] (viral load) in Plasma by Probe with signal amplification |
| 25836-8 | HIV 1 RNA [#/volume] (viral load) in Unspecified specimen by NAA with probe detection |
| 5018-7 | HIV 1 RNA [Presence] in Unspecified specimen by NAA with probe detection |
| 70241-5 | HIV 1 RNA [#/volume] (viral load) in Plasma by Probe and target amplification method detection limit = 20 copies/mL |
| 29541-0 | HIV 1 RNA [Log #/volume] (viral load) in Serum or Plasma by NAA with probe detection |
| 20447-9 | HIV 1 RNA [#/volume] (viral load) in Serum or Plasma by NAA with probe detection |
| 24013-5 | HIV 1 RNA [Interpretation] in Serum |
| 44871-2 | HIV 1 proviral DNA [Presence] in Blood by NAA with probe detection |
| 41498-7 | HIV 1 RNA [#/volume] (viral load) in Cerebral spinal fluid by NAA with probe detection |
| 48551-6 | HIV 1 RNA [#/volume] (viral load) in Serum or Plasma by Probe and target amplification method detection limit = 400 copies/mL |
| 48511-0 | HIV 1 RNA [#/volume] (viral load) in Serum or Plasma by Probe and target amplification method detection limit = 50 copies/mL |
| 41497-9 | HIV 1 RNA [Log #/volume] (viral load) in Cerebral spinal fluid by NAA with probe detection |
| 51780-5 | HIV 1 RNA [Log #/volume] (viral load) in Serum or Plasma by Probe and target amplification method detection limit = 0.5 log copies/mL |
| 48510-2 | HIV 1 RNA [Log #/volume] (viral load) in Serum or Plasma by Probe and target amplification method detection limit = 1.7 log copies/mL |
| 48552-4 | HIV 1 RNA [Log #/volume] (viral load) in Serum or Plasma by Probe and target amplification method detection limit = 2.6 log copies/mL |
| 62469-2 | HIV 1 RNA [Units/volume] (viral load) in Serum or Plasma by NAA with probe detection |
| 5017-9 | HIV 1 RNA [Presence] in Blood by NAA with probe detection |
| 50624-6 | HIV 1 RNA panel (viral load) in Cerebral spinal fluid by NAA with probe detection |
| 73658-7 | HIV 1 subtype in Unspecified specimen by NAA with probe detection |
| 53825-6 | HIV 1+Hepatitis C virus RNA [Presence] in Serum or Plasma by NAA probe detection |
| 59052-1 | HIV 1+Hepatitis C virus RNA+Hepatitis B virus DNA [Presence] in Serum or Plasma by NAA with probe detection |
| 25841-8 | HIV 2 proviral DNA [Presence] in Blood by NAA with probe detection |
| 25842-6 | HIV 2 proviral DNA [Presence] in Unspecified specimen by NAA with probe detection |
| 69354-9 | HIV 2 RNA [Units/volume] (viral load) in Serum or Plasma by NAA with probe detection |
| 69353-1 | HIV 2 RNA [Presence] in Serum or Plasma by NAA with probe detection |
| 73659-5 | HIV 2 subtype in Unspecified specimen by NAA with probe detection |
| 41516-6 | HIV 1 RNA [Log #/volume] (viral load) in Serum or Plasma by Probe with amplification detection limit = 1.9 log copies/mL |
| 41514-1 | HIV 1 RNA [Log #/volume] (viral load) in Serum or Plasma by Probe with amplification detection limit = 2.6 log copies/mL |
| 47359-5 | HIV 1 RNA [Presence] in Serum or Plasma from Donor by Probe with amplification |
| 59419-2 | HIV 1 RNA [#/volume] (viral load) in Plasma by Probe with signal amplification |
| 21008-8 | HIV 1 RNA [#/volume] (viral load) in Serum or Plasma by Probe |
| 41513-3 | HIV 1 RNA [#/volume] (viral load) in Serum or Plasma by Probe with amplification detection limit = 400 copies/mL |
| 41515-8 | HIV 1 RNA [#/volume] (viral load) in Serum or Plasma by Probe with amplification detection limit = 75 copies/mL |
| 77369-7 | HIV 1 RNA [Presence] in Semen by NAA with probe detection |
| 81246-1 | HIV 2 RNA panel – Serum or Plasma by NAA with probe detection |
| 81652-0 | HIV 2 RNA [Log #/volume] (viral load) in Serum or Plasma by NAA with probe detection |
| 85361-4 | HIV 1+2 RNA [Presence] in Blood by NAA with probe detection |
| 85368-9 | HIV 1+2 RNA [Presence] in Serum or Plasma by NAA with probe detection |
| 86547-7 | HIV 2 RNA [#/volume] (viral load) in Cerebral spinal fluid by NAA with probe detection |
| 86548-5 | HIV 2 RNA [#/volume] (viral load) in Plasma by NAA with probe detection |
| 86549-3 | HIV 2 RNA [Log #/volume] (viral load) in Cerebral spinal fluid by NAA with probe detection |
| 25835-0 | HIV 1 RNA [Presence] in Serum or Plasma by NAA with probe detection |

**Appendix C**

**Antiretroviral Medications Used for HIV Treatment**

Table 3 provides a list of generic and brand names for the antiretroviral medications used for the treatment of HIV.

| **Table 3: Antiretroviral Medications for HIV Treatment** | |
| --- | --- |
| **Generic Name (Abbreviation)** | **Brand Name** |
| Emtricitabine (FTC) | Emtriva |
| Lamivudine (3TC) | Epivir |
| Zalcitabine, dideoxycytidine (ddC) | Hivid |
| Zidovudine, azidothymidine (AZT, ZDV) | Retrovir |
| Didanosine, dideoxyinosine (ddI) | Videx |
| Tenofovir disoproxil fumarate (TDF) | Viread |
| Stavudine (d4T) | Zerit |
| Abacavir sulfate (ABC) | Ziagen |
| Rilpivirine | Edurant |
| Etravirine | Intelence |
| Delaviridine (DLV) | Rescriptor |
| Efavirenz (EFV) | Sustiva |
| Nevirapine (NVP) | Viramune |
| Amprenavir (APV) | Agenerase |
| Tipranavir (TPV) | Aptivus |
| Indinavir (IDV) | Crixivan |
| Saquinavir | Fortovase |
| Saquinavir mesylate (SQV) | Invirase |
| Fosamprenavir calcium (FOS-APV) | Lexiva |
| Ritonavir (RTV) | Norvir |
| Darunavir | Prezista |
| Atazanavir sulfate (ATV) | Reyataz |
| Nelfinavir mesylate (NFV) | Viracept |
| Enfuvirtide (T-20) | Fuzeon |
| Maraviroc | Selzentry |
| Raltegravir | Isentress |
| Dolutegravir | Tivicay |
| Elvitegravir | Vitekta |
| Cobicistat | Tybost |
| Efavirenz/emtricitabine/tenofovir disoproxil fumarate | Atripla |
| Emtricitabine/rilpivirine/tenofovir disoproxil fumarate | Complera |
| Atazanavir/cobicistat | Evotaz |
| Cobicistat/darunavir ethanolate | Prezcobix |
| Elvitegravir/cobicistat/emtricitabine/tenofovir disoproxil fumarate | Stribild |
| Lamivudine/zidovudine | Combivir |
| Abacavir/lamivudine | Epzicom |
| Abacavir/zidovudine/lamivudine | Trizivir |
| Tenofovir disoproxil fumarate/emtricitabine | Truvada |
| Lopinavir/ritonavir | Kaletra |
| Tenofovir alafenamide/emtricitabine | Descovy |
| Rilpivirine/tenofovir disoproxil fumarate/emtricitabine | Odefsey |
| Elvitegravir/cobicistat/tenofovir alafenamide/emtricitabine | Genvoya |
| Dolutegravir/abacavir/lamivudine | Triumeq |

**Appendix D**

**Medications for HIV Pre-exposure prophylaxis (PrEP) and Hepatitis B (HBV) Infection**

Some antiretroviral medications used to treat HIV can also be prescribed for HIV prevention (PrEP) or to treat HBV infection. Patients prescribed any of the medications listed in Table 4, in the absence of prescriptions for any of the HIV-specific antiretrovirals listed in Appendix C, and in the absence of a positive HIV confirmatory test and a positive HIV viral load (Appendix A and B), should be excluded from the cohort of people living with HIV.

| **Table 4: Antiretrovirals used for PrEP and/or HBV treatment** | | |
| --- | --- | --- |
| **Indication** | **Generic Name (Abbreviation)** | **Brand Name** |
| PrEP/HBV | Emtricitabine (FTC) | Emtriva |
| HBV | Lamivudine (3TC) | Epivir |
| PrEP/HBV | Tenofovir disoproxil fumarate (TDF) | Viread |
| PrEP/HBV | Tenofovir disoproxil fumarate/emtricitabine | Truvada |
| PrEP | Tenofovir alafenamide/emtricitabine | Descovy |
